# Asymptomatic Cervical Cancer Recurrence Presenting As Gastric Metastasis: A Report of a Rare Case

**DOI:** 10.7759/cureus.102001

**Published:** 2026-01-21

**Authors:** Vaibhav Sharma, Nikita Gupta, Paul D Leger, William T Ryan, Sonali Rudra

**Affiliations:** 1 Department of Radiation Medicine, MedStar Georgetown University Hospital, Washington, DC, USA; 2 Department of Oncology, MedStar Georgetown University Hospital, Washington, DC, USA; 3 Department of Pathology, MedStar Georgetown University Hospital, Washington, DC, USA

**Keywords:** asymptomatic recurrence, cervical cancer, gastric metastasis, gastric outlet obstruction, oligometastatic disease, palliative radiotherapy, pembrolizumab, squamous cell carcinoma

## Abstract

Gastric metastasis from cervical cancer is exceedingly rare, with limited data to guide diagnosis and management. Reported cases are typically associated with widespread disease and poor prognosis. We present the case of a 56-year-old woman with stage IIIA squamous cell carcinoma of the cervix. She was initially treated with cisplatin-based chemoradiation followed by interstitial brachytherapy. Surveillance imaging identified a new gastric antral mass. Endoscopic biopsy confirmed metastatic squamous cell carcinoma consistent with the cervical primary. PET/CT revealed isolated gastric and right parametrial involvement. She was started on carboplatin and paclitaxel, but progressed to partial gastric outlet obstruction. She subsequently received palliative external beam radiation to the gastric lesion (37.5 Gy in 15 fractions) and was started on pembrolizumab based on strong programmed death-ligand 1 (PD-L1) expression (combined positive score (CPS) of 100). Despite treatment, she experienced further clinical and radiographic progression. Her condition continued to worsen, leading to hospitalization with multiple complications. She ultimately succumbed to her illness approximately one year after diagnosis. This case highlights the clinical challenges of managing gastric metastasis as a site of recurrence following definitive treatment for cervical cancer. Although a multimodal approach incorporating chemotherapy, radiation, and immunotherapy was employed, the patient experienced disease progression. Early detection and symptom-directed local therapies may offer temporary benefit; however, outcomes remain poor, and data on overall survival in this setting are extremely limited. As systemic therapies improve survival in advanced cervical cancer, awareness of atypical metastatic patterns such as gastric involvement will become increasingly important in guiding surveillance and treatment strategies.

## Introduction

Cervical cancer is a common cause of gynecologic malignancy worldwide, with 13,360 new cases estimated for 2025 [1]. Squamous cell carcinoma is far more common than adenocarcinoma of the cervix, which is also frequently due to high-risk human papillomavirus (HPV) [2]. The most common metastatic sites include the lungs, liver, and bone, with prognosis generally poor once distant spread occurs [3]. Gastric metastases are particularly rare, reported in approximately 0.2-0.8% of necropsies in the general population and 1.7-5.4% of patients with known malignancy, with the lung, breast, melanoma, and esophagus representing the most common primary sites; gastric involvement from cervical cancer remains uncommon, described primarily in small series and isolated case reports [4]. Such patients may initially be asymptomatic or have vague, nonspecific symptoms, with more overt complaints such as dysphagia or hematemesis typically emerging only once the gastric lesion becomes advanced, resulting in delayed or incidental diagnosis. In this case report, we present an asymptomatic gastric recurrence discovered incidentally on a surveillance CT scan. We also outline known cases of gastric metastases from cervical cancer, focusing on disease-free interval, anatomic region of recurrence, diagnostic methods, clinical outcomes, and treatment considerations.

## Case presentation

The patient is a 56-year-old premenopausal female, gravida 1 para 1 (G1P1), with a history of stage IIIA cervical cancer diagnosed in late 2022 after presenting with vaginal bleeding. She initially reported three months of heavy menstrual bleeding prior to presentation. On bimanual and rectovaginal examination, the uterus was enlarged and globular (approximately 10-12 cm) with a firm 4-5 cm cervical mass extending from the 3 o’clock to the 9 o’clock positions and involving the posterior vagina, without palpable adnexal masses or rectal nodularity. Biopsy of the lesion revealed moderately differentiated squamous cell carcinoma of the cervix. Although radical hysterectomy with bilateral salpingo-oophorectomy was initially planned, the procedure was aborted intraoperatively when examination under anesthesia and exploratory findings revealed a 4-5 cm cervical mass with concern for extension into the lower third of the vagina, rendering the tumor surgically unresectable. Pelvic MRI subsequently demonstrated a 7.8 cm heterogeneously enhancing cervical mass extending into the upper two-thirds of the vagina with lateral extension beyond the cervical stroma into the parametrium, without involvement of the lower one-third of the vagina, pelvic wall, bladder, or rectum. PET/CT demonstrated a hypermetabolic lymph node within the left inguinal region. Treatment consisted of cisplatin 50 mg/m2 every three weeks for six cycles [5] combined with intensity-modulated radiation therapy delivering 45 Gy to the pelvis with a simultaneous integrated boost to 55 Gy in 25 fractions to a PET-avid inguinal lymph node [5]. Given persistent bulky disease on follow-up MRI, this was followed by an interstitial brachytherapy boost of 5.5 Gy in five fractions to the primary tumor, completed in early 2023. Her first PET/CT following treatment showed no evidence of metabolic activity in the primary tumor, but there was uptake in the lung (which was also present in her initial staging PET/CT and initially thought to be infectious/inflammatory in nature). Repeat PET/CT revealed a slight increase in pulmonary uptake. Subsequent biopsy was consistent with cryptogenic organizing pneumonia, treated with prednisone starting in September 2023.

**Figure 1:**
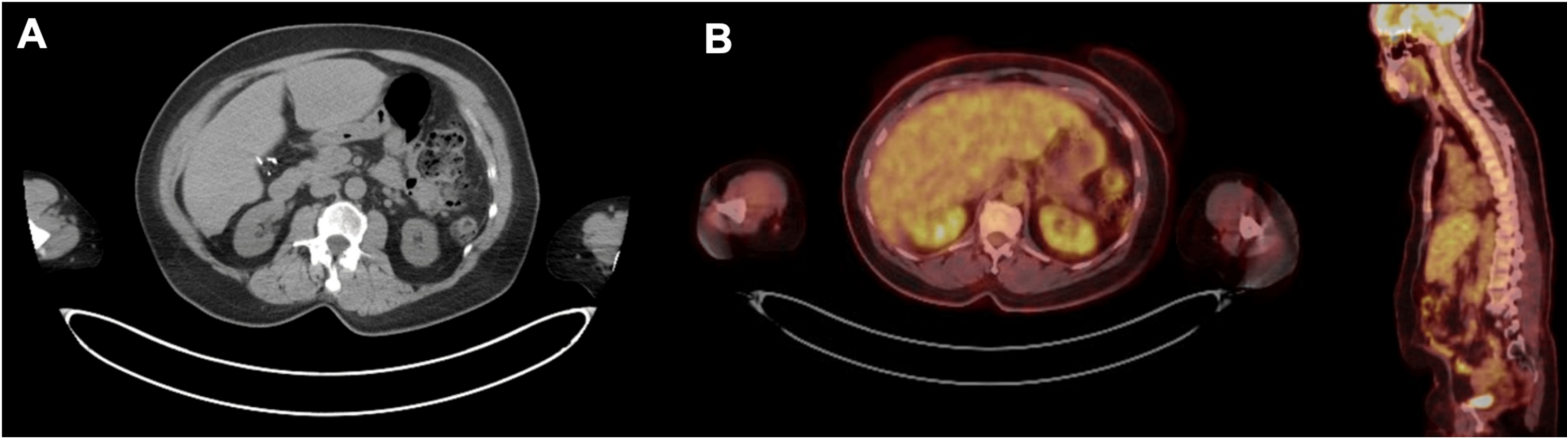
Routine surveillance imaging from January 2024 demonstrating no gastric involvement (A) Axial non-contrast CT of the abdomen showing no focal gastric wall thickening or mass. (B) Axial PET/CT of the abdomen demonstrating no hypermetabolic gastric activity. All images are from the same imaging time point and show no evidence of gastric metastasis.

**Figure 2:**
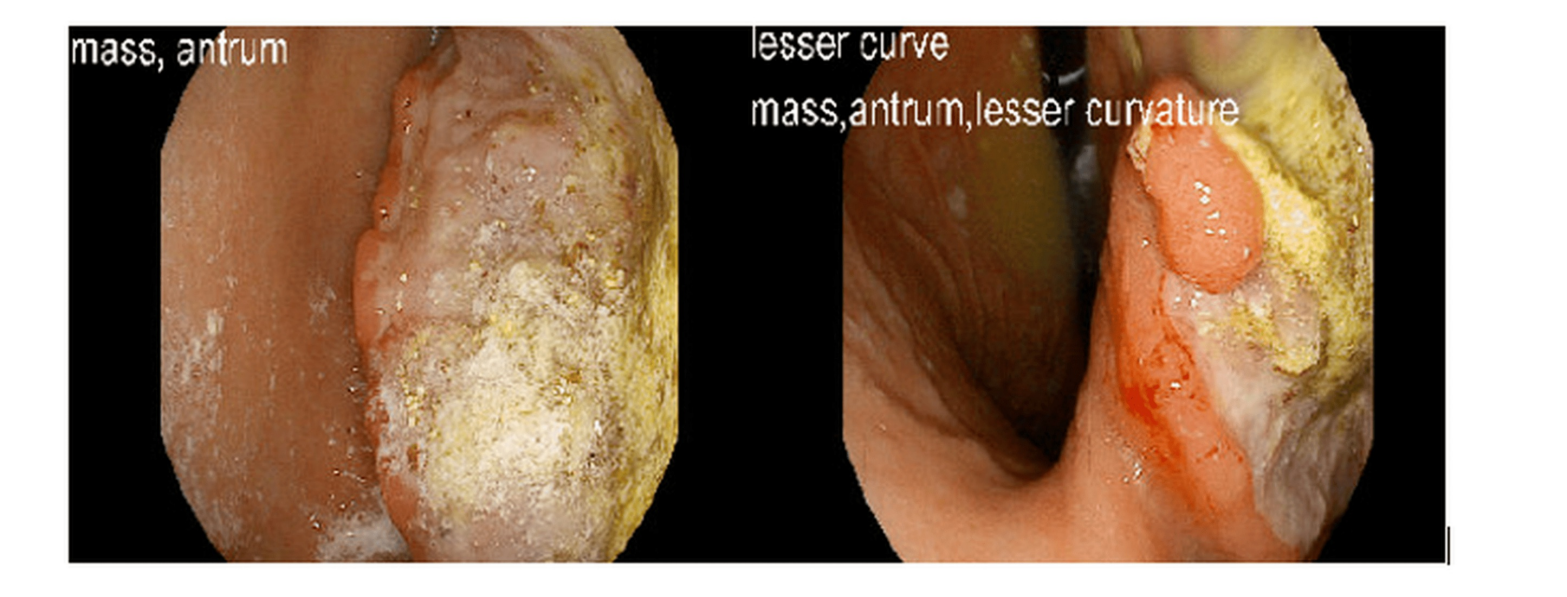
Endoscopic appearance of a gastric antral metastasis along the lesser curvature Esophagogastroduodenoscopy images demonstrating an ulcerated, infiltrative mass in the gastric antrum (left panel, “mass, antrum”) and extension of the lesion along the lesser curvature of the antrum (right panel, “mass, antrum, lesser curvature”). The lesion occupied a substantial portion of the antral lumen and was biopsied. Histopathology confirmed metastatic squamous cell carcinoma consistent with the patient’s known cervical primary.

**Figure 3:**
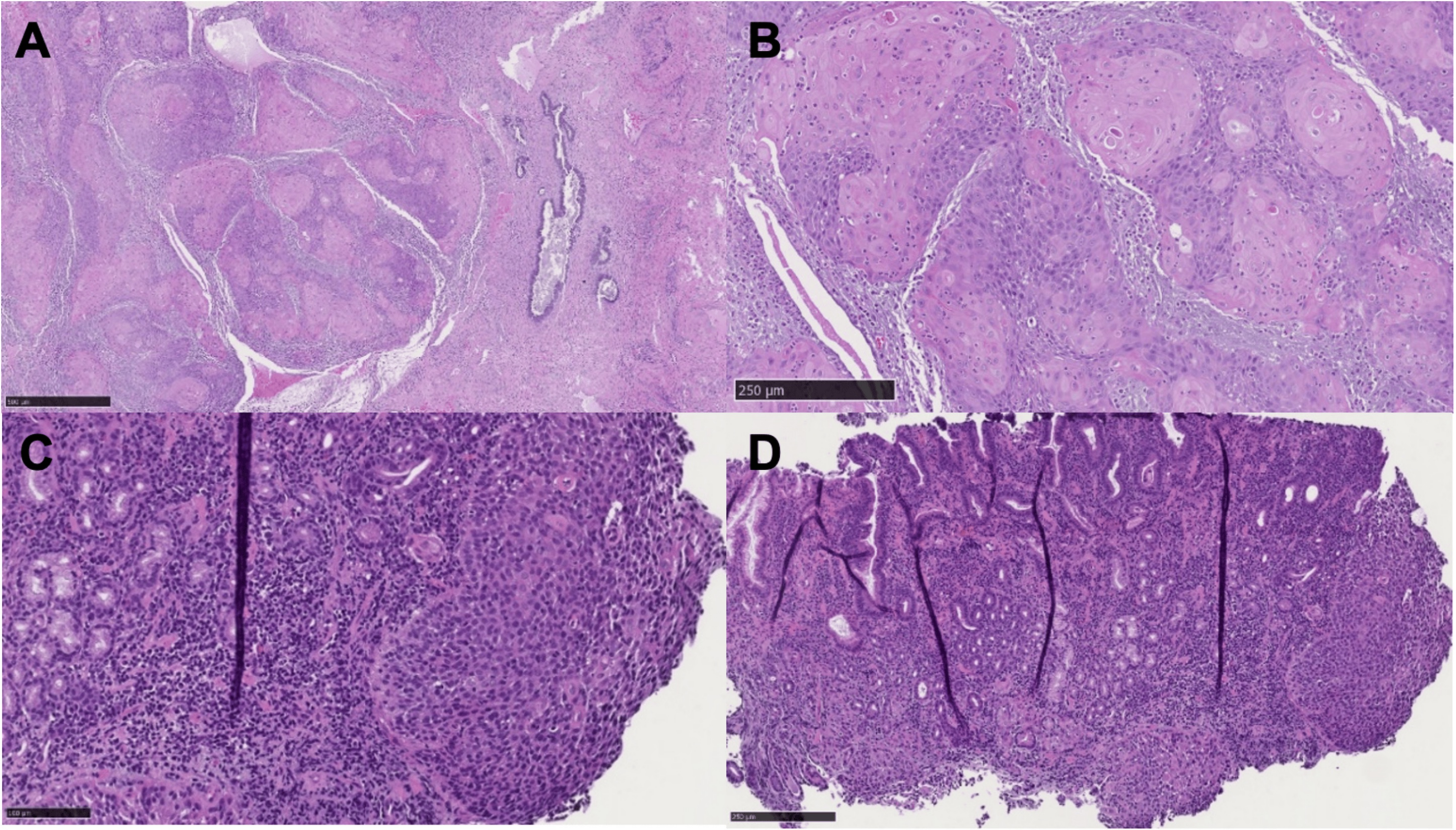
Histological comparison of the primary cervical tumor and gastric metastasis Representative histological images of (A) moderately differentiated squamous cell carcinoma of the cervix with normal gland (5×), (B) moderately differentiated squamous cell carcinoma (7.59×), (C) normal stomach (10×), and (D) gastric tissue with recurrent squamous cell carcinoma (20×).

**Figure 4:**
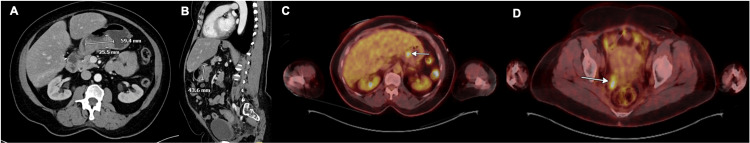
Imaging demonstrating interval development of gastric recurrence (A) Axial contrast-enhanced CT of the abdomen showing focal mural thickening of the posterior gastric antrum, concerning for recurrent disease. (B) Sagittal contrast-enhanced CT of the abdomen further delineates the antral lesion. (C) Axial PET/CT of the abdomen demonstrating corresponding hypermetabolic activity within the gastric antrum (arrow). (D) Axial PET/CT of the pelvis showing hypermetabolic soft tissue in the right parametrial region, near the primary site of cervical disease (arrow). Images represent a subsequent imaging time point, prompting further evaluation with endoscopy and biopsy.

In follow-up one year after chemoradiation, the patient developed constipation and mild rectal bleeding and was referred to gastroenterology. Colonoscopy in January 2024 revealed persistent non-malignant radiation changes. Her PET/CT in January 2024 was negative for any abnormality, and a repeat PET/CT in six months was ordered to confirm stability. In August 2024, she underwent a routine CT of the chest, abdomen, and pelvis, which identified focal mural thickening in the posterior gastric antrum (Figure 1). A subsequent endoscopy revealed a mass in the stomach involving 40% of the lumen in the pre-pyloric antrum and extending into the lesser curvature (Figure 2). A biopsy confirmed moderately-differentiated squamous cell carcinoma with immunohistochemical features consistent with the patient’s known primary cervical carcinoma (Figure 3). Immunohistochemical profiling supported metastatic disease from the known cervical primary, with tumor cells positive for p40 and p63, consistent with squamous differentiation [6], and diffuse p16 overexpression, supporting an HPV-associated cervical squamous cell carcinoma [7]. The tumor was negative for CK7 and CK20, arguing against a primary gastric adenocarcinoma, which typically demonstrates CK7 positivity with variable CK20 expression [8]. Taken together, the immunophenotype favored gastric metastasis from cervical squamous cell carcinoma rather than a primary gastric malignancy. She then underwent PET/CT, which confirmed a hypermetabolic lesion in the posterior gastric antrum alongside soft tissue thickening, as well as a right parametrial region of hypermetabolic activity near the primary site of cervical disease (Figure 4). These findings illustrate the transition from no visible gastric disease on surveillance imaging to biopsy-confirmed gastric metastasis within an eight-month interval. At the time of the esophagogastroduodenoscopy (EGD), the patient was relatively asymptomatic and only reported mild fatigue and constipation. However, the complete blood count (CBC) showed a hemoglobin of 5.2 g/dL. She subsequently was found to have recurrent transfusion-dependent anemia. There was a discussion of whether to start treatment with chemotherapy or surgery, given her limited sites of disease and the transfusion-dependent nature of the gastric lesion. Ultimately, the decision was made to proceed with chemotherapy first since the parametrial disease was in the prior radiation field and would increase the morbidity of surgery.

**Figure 5:**
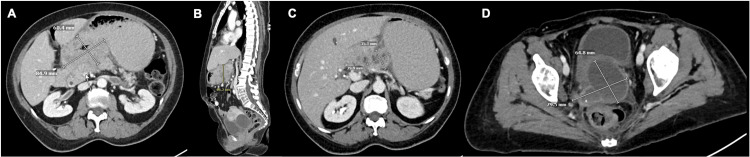
Post-chemotherapy imaging demonstrating marked disease progression (A) Axial contrast-enhanced CT of the abdomen showing significant interval enlargement of the distal gastric mass (8.5 × 6.0 cm; previously 7.0 × 3.0 cm), now confluent with enlarged gastrohepatic nodal metastases. (B) Sagittal contrast-enhanced CT of the abdomen demonstrating loss of the fat plane between the gastric mass and pancreas, concerning for direct invasion. (C) Axial contrast-enhanced CT of the abdomen showing marked interval enlargement of gastrohepatic lymph node metastases. (D) Axial contrast-enhanced CT of the pelvis demonstrating interval growth of the pelvic mass involving the uterine cervix. These images were obtained at a later imaging time point than those shown in Figures 2-3.

The patient was initiated on carboplatin and paclitaxel, completing five of six planned cycles. The fifth cycle was dose-reduced due to treatment-related toxicity. Her course was complicated by pancytopenia, leading to delays in chemotherapy administration, as well as transfusion-dependent anemia requiring near-weekly red blood cell transfusions. Restaging imaging revealed significant disease progression, including marked enlargement of the gastric mass with loss of separation from gastrohepatic nodal metastases, obliteration of the fat plane with the pancreas concerning for direct invasion, and a substantially enlarged pelvic mass (Figure 5). She also developed partial gastric outlet obstruction, contributing to worsening symptoms of nausea, vomiting, and poor oral intake. She was hospitalized in March 2025 with a hemoglobin of 5.8 g/dL on admission and poorly controlled pain. Initially, expedited surgery was considered, given the growth of the lesion, ongoing bleeding, and symptom burden. However, the multidisciplinary tumor board decision was to proceed with non-operative management with radiation therapy. She received palliative external beam radiation therapy to a total dose of 37.5 Gy in 15 fractions. CARIS molecular profiling revealed strong PD-L1 expression with a combined positive score (CPS) of 100. She was then started on adjuvant pembrolizumab. Despite receiving two cycles of pembrolizumab, the patient developed worsening gastric outlet obstruction, symptomatic anemia, and evidence of progressive disease. Imaging revealed interval growth of hepatic metastases, pelvic mass enlargement, and a stable but infiltrative gastric mass. Her hospitalization was marked by several complications, including pancytopenia with superimposed infection necessitating intravenous antibiotics and antifungal therapy. She also underwent placement of a biliary drain by interventional radiology for a perigastric fluid collection. Despite these interventions, her condition progressively worsened, requiring transfer to the medical intensive care unit for hypoxic and hypercapnic respiratory failure, and she ultimately succumbed to her illness 11 days after ICU transfer.

## Discussion

Gastric metastasis from cervical cancer is extremely rare and often goes undetected until symptoms such as anemia, melena, or obstruction prompt further evaluation. Our review of the literature yielded only six reported cases. See Table 1 for a summary of reported cases of gastric metastasis from cervical cancer, including presentation, diagnostics, salvage treatment, and outcomes. Most cases are identified in the context of diffuse metastatic disease and are associated with poor outcomes. However, a smaller subset of patients presents with isolated or oligometastatic gastric involvement, in whom more aggressive local therapies may provide meaningful clinical benefit.

**Table 1:** Summary of reported cases of gastric metastasis from cervical cancer in the literature Cases are compared by age at diagnosis, initial pathology, initial treatment, time to recurrence, pattern of failure, diagnostic imaging modality, location of gastric metastasis, metastatic pathology, salvage treatment, and clinical outcome. CT: computed tomography; MRI: magnetic resonance imaging; EGD: esophagogastroduodenoscopy; PET-CT: positron emission tomography-computed tomography; SCC: squamous cell carcinoma; GAS: gastric-type adenocarcinoma; HPV: human papillomavirus; HDR: high-dose-rate; Gy: gray; 5-FU: 5-fluorouracil

Cases	Initial Age at Presentation	Initial Pathology and Treatment	Time Between Gastric Mets Presentation	Pattern of Failure (Local vs. Oligo vs. Diffuse Mets)	Diagnostic Imaging Modality Used	Location	Metastatic Pathology	Salvage Treatment	Clinical Outcome at Time of Publication
A rare case of gastric squamous cell carcinoma metastasized from the cervix (Oriuchi et al.) [9]	59	Cervical SCC	At the time of diagnosis	Diffuse	EGD, PET-CT, CT, MRI	Body and antrum	SCC	Palliative care	Died within two months of stage IV diagnosis
Gastric metastasis of cervical carcinoma, rare cause of gastrointestinal bleeding (Simões et al.) [10]	43	Cervical SCC: Treated with initial chemotherapy and radiation therapy.	Three years	Diffuse	Endoscopy, CT	Cardia	Cervical SCC	Palliative chemotherapy	Died in hospital, 19 days after diagnosis of metastatic disease
Gastric and colonic metastasis from cancer cervix: an unusual progression with an uncommon cause of mortality (Singhal et al.) [11]	48	Well-differentiated SCC of the ectocervix: Treated with total hysterectomy.	Six years (colonic metastasis), eight years (gastric metastasis)	Diffuse	CT, colonoscopy	Fundus and body	SCC with colonic and gastric metastasis	Segmental resection, six cycles of cisplatin and 5-FU, palliative chemo (cisplatin, paclitaxel, 5-FU)	Death before planned palliative surgery due to massive gastric bleeding
Gastric metastasis from cervical cancer (Dhanushkodi et al.) [12]	38	Adeno-squamous carcinoma, stage IIB: Treated with 50 Gy pelvic radiation with chemotherapy and HDR intracavitary application.	Seven months	Diffuse	CT, endoscopy	Antrum and body	Adeno-squamous carcinoma	Palliative chemotherapy	Alive, treated with palliative chemotherapy
Gastric metastasis of cervix uteri carcinoma, rare cause of lower gastric stenosis (Moldovan et al.) [13]	63	SCC of cervix: Treated with surgery, chemotherapy, and radiation therapy.	Two-year severe pyloric stenosis presentation	Oligometastatic	Endoscopy, abdominal MRI	Antrum	Intramural antropyloric SCC	Subtotal gastrectomy with lymphadenectomy, omentectomy, hepatectomy of segments 4-5, 6 cycles of paclitaxel-carboplatin chemotherapy, 45 Gy radiation	Alive 15 months after surgery without progression
Gastric metastasis from gastric-type mucinous adenocarcinoma of uterine cervix: a case report (Kim et al.) [14]	61	Endometrioid carcinoma with cervical invasion: Treated with total hysterectomy, bilateral salpingo-oophorectomy, and pelvic and para-aortic lymph node dissection.	At the time of initial diagnosis	Diffuse	CT, initial contrast-enhanced pelvic MRI, abdominal CT, endoscopy	Antrum	HPV-independent GAS	Six cycles of paclitaxel-cisplatin-avastin chemotherapy followed by pembrolizumab	Progression noted 12 months after chemotherapy; palliative treatment after suspected peritoneal seeding

Patterns of gastric metastasis from cervical cancer

Diffuse or Multifocal Involvement

In the majority of published cases, gastric metastases arise as part of widespread disease dissemination and are associated with limited survival. Oriuchi et al. described a 59-year-old woman who presented with anorexia and was found to have multiple submucosal gastric lesions on EGD. Biopsy confirmed squamous cell carcinoma, and PET/CT demonstrated hypermetabolic lesions in the stomach, uterus, and spine. Due to poor performance status, she was not a candidate for systemic therapy and died within two months of diagnosis [9]. Similarly, Simões et al. reported a 43-year-old woman who developed anemia, melena, and weight loss three years after treatment for cervical SCC. Endoscopy revealed a friable, ulcerated mass at the gastric cardia, and biopsy confirmed metastatic disease. Despite plans for palliative chemotherapy, she rapidly deteriorated and died in hospital, 19 days after her diagnosis [10]. These cases underscore the aggressive behavior of cervical cancer following extra-pelvic dissemination, particularly with rare involvement of the upper gastrointestinal (GI) tract.

Other reports describe GI metastases that are multifocal or involve multiple segments of the GI tract. Singhal et al. described a woman with a remote history of hysterectomy and bilateral salpingo-oophorectomy (removal of the uterus, cervix, and both fallopian tubes and ovaries) for cervical SCC who initially developed colonic obstruction due to metastatic disease, which was treated with segmental resection and colo-colic anastomosis and six cycles of cisplatin and 5-fluorouracil (5-FU). She then presented with gastric metastasis two years later, in the lesser sac, pancreatic tail, and spleen. Despite an initial response to systemic therapy, she ultimately died from massive GI bleeding before planned palliative surgery [11]. Similarly, Dhanushkodi et al. reported a patient who developed gastric metastasis seven months after chemoradiation for stage IIIB adenosquamous carcinoma, with tumor infiltrating into the transverse colon, precluding surgical intervention [12]. These cases collectively illustrate that gastric metastases from cervical cancer, whether presenting diffusely or as part of multifocal GI involvement, tend to exhibit aggressive behavior and are frequently associated with poor outcomes. Response to systemic chemotherapy remains limited.

Isolated or Oligometastatic Involvement

In contrast to widespread disease, a small number of cases describe isolated or oligometastatic gastric involvement with more favorable outcomes. Moldovan et al. presented a 49-year-old woman who developed symptoms of gastric outlet obstruction two years after treatment for stage IIB cervical SCC. Imaging and endoscopy revealed a circumferential lesion in the gastric antrum causing pyloric stenosis. She underwent D2 subtotal gastrectomy with lymphadenectomy and omentectomy. Pathology confirmed a solitary gastric metastasis without mucosal involvement and one positive perigastric lymph node. Postoperative chemotherapy and 45 Gy of radiation were delivered, and the patient remained disease-free at 15-month follow-up [13]. Kim et al. reported a unique case of gastric-type mucinous adenocarcinoma presenting with synchronous cervical and gastric lesions. The patient underwent subtotal gastrectomy, hysterectomy, bilateral salpingo-oophrectomy, and pelvic and para-aortic lymph node dissection followed by systemic therapy. She achieved temporary disease control for 12 months, after which she was started on pembrolizumab due to small bowel and peritoneal seeding [14]. These cases suggest that in patients with isolated or limited metastatic spread, especially with delayed recurrence or long disease-free intervals, aggressive local treatment such as surgery or radiation may offer control.

Treatment selection based on disease extent

Role of Salvage Chemotherapy

In published case reports, chemotherapy has often been used in neoadjuvant, adjuvant, or palliative settings for cervical cancer with gastric metastases. However, its role must be carefully individualized based on the extent of disease, patient performance status, and symptom burden. In cases of disseminated disease or when micrometastatic control is needed, chemotherapy can provide systemic benefit and serve as a bridge to further treatment. Conversely, in patients with active bleeding, obstruction, or rapid clinical decline, systemic therapy alone is often insufficient, and early integration of local measures may be more effective.

As previously described by Simões et al., rapid disease progression can outpace systemic treatment planning, ultimately preventing the initiation of chemotherapy. Moldovan et al. detailed a patient with a solitary antral lesion and outlet obstruction treated with subtotal gastrectomy followed by adjuvant chemotherapy and radiation, achieving 15 months of disease-free survival, demonstrating optimal use of systemic therapy in conjunction with local control [13]. In contrast, Dhanushkodi et al. reported a case of diffuse gastric and colonic involvement just seven months after chemoradiation for stage IIB adenosquamous carcinoma. The patient was not a surgical candidate and was managed with palliative chemotherapy alone [12]. Though alive at follow-up, her case reflects the limited long-term control achievable with systemic therapy alone in the setting of multifocal GI metastases.

In our case, chemotherapy was initiated following the diagnosis of metastatic gastric involvement, but was complicated by pancytopenia and transfusion-dependent anemia, requiring early dose reductions. Continued disease progression and declining performance status ultimately limited further systemic options. This clinical course prompted the integration of palliative radiation for local control and immunotherapy for systemic disease. These cases collectively underscore that while chemotherapy is widely employed, its utility as a stand-alone therapy is limited in the presence of bulky, symptomatic gastric disease. Early multidisciplinary planning is essential to determine when chemotherapy is most beneficial, typically in patients with preserved marrow function, minimal symptoms, and limited local tumor burden, and when prompt local or immunologic intervention should take precedence.

Role of Salvage Surgery and Radiation

Local treatment has an important role in managing gastric metastases from cervical cancer, particularly in patients who present with symptomatic disease such as bleeding, obstruction, or rapid clinical decline. In select patients with limited burden of disease, aggressive local therapy may offer durable control or palliation. The decision to use surgery, radiation, or a combination of both is guided by the patient’s functional status, comorbidities, disease distribution, and symptom severity.

As previously discussed, Moldovan et al. reported a solitary antral lesion treated with subtotal gastrectomy and adjuvant therapy, resulting in 15 months of disease-free survival [13]. This case illustrates the importance of timely local intervention. The timing of local therapy was crucial, as surgery addressed mechanical obstruction and enabled nutritional recovery, while adjuvant therapy targeted residual microscopic disease. In the previously cited case by Singhal et al., delayed gastrectomy contributed to fatal hemorrhage, emphasizing the risks of deferring local intervention [11].

In our case, the patient presented with transfusion-dependent bleeding and partial gastric outlet obstruction. Due to poor surgical candidacy and chemotherapy-related cytopenias, we opted for external beam radiation to the gastric lesion. Disease progression during systemic therapy further limited surgical options and made the timing of local intervention more challenging. The patient’s declining functional reserve and expanding disease burden necessitated a less invasive approach. Radiation provided effective hemostasis and symptom control, highlighting its utility in palliative settings for patients unable to tolerate surgery. Radiotherapy also avoids the morbidity associated with gastrectomy. Although data remain limited, these cases support the integration of local therapy when symptoms are not adequately addressed by chemotherapy alone. The timing of intervention must be carefully considered, as delaying local treatment in symptomatic patients can lead to further clinical deterioration and loss of opportunity for curative or stabilizing approaches.

Role of Immunotherapy

The introduction of immune checkpoint inhibitors has significantly transformed the treatment landscape for advanced cervical cancer. Pembrolizumab, an anti-PD-1 antibody, has demonstrated improved progression-free survival (10.4 vs. 8.2 months) and overall survival (OS) (53.0% vs. 41.7% at 24 months) in PD-L1-positive patients with persistent, recurrent, or metastatic cervical cancer receiving platinum-based chemotherapy, as shown in the phase III KEYNOTE-826 trial [15]. Additionally, the phase III KEYNOTE-A18 trial showed that the addition of pembrolizumab to concurrent chemoradiotherapy significantly improved 24-month progression-free survival (68% vs. 57%) in patients with newly diagnosed, high-risk, locally advanced disease [16]. Other agents such as cemiplimab (EMPOWER-Cervical 1; median OS 12.0 vs. 8.5 months) and tisotumab vedotin (GOG-3016; 24% response rate) have expanded systemic treatment options in the second-line setting [17,18]. In the only other reported case involving gastric metastases, Kim et al. described a patient with gastric-type mucinous adenocarcinoma who underwent surgery, chemotherapy, and was subsequently treated with pembrolizumab for suspected peritoneal seeding; the patient was alive at the time of publication [14]. In our case, pembrolizumab was initiated following limited chemotherapy benefit and hematologic toxicity. Despite a high PD-L1 CPS of 100, the patient experienced rapid clinical and radiographic progression following two cycles, with worsening gastric outlet obstruction, anemia, hepatic and pelvic disease progression, and vascular involvement. This case illustrates the limited efficacy of immunotherapy in select cases of recurrent metastatic cervical cancer following definitive treatment and highlights the need for more robust predictive biomarkers and prospective studies to better guide patient selection, particularly in the setting of rare metastatic sites such as gastric involvement.

Implications for surveillance and management

Standard surveillance for cervical cancer includes a PET/CT or MRI performed three to six months after completion of chemoradiation. Thereafter, routine follow-up consists of physical examinations alone, with imaging reserved for patients who develop symptoms or have clinical suspicion for recurrence. As a result, gastric metastases may remain undetected until they become symptomatic or reach an advanced stage. In oligometastatic cases, where local therapy may provide temporary clinical benefit, broader surveillance strategies could facilitate earlier detection of isolated lesions and allow for more timely intervention.

As more patients undergo multimodal therapy and survive longer, rare patterns of recurrence, such as gastric involvement, may become more clinically relevant. Further research is needed to define which patients are most likely to benefit from routine surveillance and aggressive local therapy in the setting of gastric metastasis from cervical cancer.

## Conclusions

Gastric metastasis from cervical cancer is rare and typically signals advanced disease. While outcomes are often poor, select patients may benefit from a multimodal approach. Chemotherapy can provide systemic control but is often limited by toxicity, particularly in symptomatic patients. Local therapies such as surgery or radiation play a critical role in managing bleeding or obstruction, while immunotherapy has shown promise in PD-L1-positive cervical cancer in other clinical contexts. Our patient initially presented with asymptomatic gastric involvement and limited sites of disease but progressed despite chemotherapy and required palliative radiation for bleeding and obstruction. Immunotherapy was given for PD-L1 positivity, but the disease progressed. Although this multimodal approach did not achieve durable disease control, it may have temporarily slowed progression and alleviated symptoms; however, her condition worsened, and she ultimately succumbed to her illness approximately one year after diagnosis of gastric metastasis. This case highlights the clinical challenges associated with gastric metastases in advanced cervical cancer and emphasizes the need for more effective therapies and refined approaches to patient selection in this rare metastatic context. To our knowledge, this is the first reported case of gastric metastasis from cervical cancer that initially presented asymptomatically in the setting of recurrent, low-volume disease following definitive chemoradiation, and was subsequently managed with salvage chemotherapy, palliative gastric radiation, and adjuvant pembrolizumab.
